# Emergency departments as under-utilized venues to provide HIV prevention services to female sex workers in Nairobi, Kenya

**DOI:** 10.1186/s12245-023-00516-x

**Published:** 2023-08-03

**Authors:** Amanda Poxon, Maria Leis, Miranda McDermott, Antony Kariri, Rupert Kaul, Joshua Kimani

**Affiliations:** 1 Department of Medicine, University of Toronto, 1 King’s College Circle, Toronto, M5S1A8 Canada; 2 Department of Medical Microbiology, University of Nairobi, Nairobi, Kenya; 3 Department of Medical Microbiology, University of Manitoba, Manitoba, Canada

**Keywords:** Female sex workers, Emergency medicine, Acute care, HIV

## Abstract

**Background:**

Female sex workers (FSW) in sub-Saharan Africa are disproportionately affected by HIV and remain a key target population for efforts to reduce transmission. While HIV prevention tools such as PEP and PrEP are available through outpatient FSW clinics, these services are underused. Emergency medicine is a rapidly expanding field in Kenya and may provide a novel venue for initiating or optimizing HIV prevention services. This study examined the characteristics of FSW from Nairobi, Kenya, who had utilized an emergency department (ED) during the past year to broaden our understanding of the patient factors related to usage.

**Methods:**

An anonymous questionnaire was administered to a convenience sample of 220 Nairobi FSW attending dedicated clinics from June to July 2019. The participants were categorized into those who attended an ED over the past year (acute care users) and clinic-only users (control). A modified version of the WHO Violence Against Women Instrument assessed gender-based violence. Multivariable negative binomial logistic regressions evaluated predictors of health care use among these populations.

**Results:**

Of the total 220 women (median [IQR] age 32 [27–39]), 101 and 116 were acute care and control populations, respectively. Acute care users had 12.7 ± 8.5 healthcare visits over a 12-month period, and the control population had 9.1 ± 7.0 (*p* < 0.05). ED attendance did not improve the PrEP usage, with 48.5%, and 51% of acute care and clinic users indicated appropriate PrEP use. Patient factors that correlated with health care utilization among acute care users included client sexual violence (OR 2.2 [1.64–2.94], *p* < 0.01), PrEP use (OR 1.54 (1.25–1.91), < 0.01), and client HIV status (OR 1.35 (1.02–1.69), *p* < 0.01).

**Conclusions:**

Many FSW at high risk for HIV were not accessing HIV prevention tools despite attending a dedicated FSW clinic offering such services. FSW who had attended an ED over the past year had a higher prevalence of HIV risk factors, demonstrating that emergency departments may be important acute intervention venues to prevent HIV transmission in this population. These results can guide policy design, health care provider training, and facility preparedness to support strategies aimed at improving HIV prevention strategies for FSW in Kenyan ED’s.

## Background

The prevalence of HIV among female sex workers (FSW) in sub-Saharan Africa is disproportionately high, estimated to be between 24 and 72% in some regions [1, 2]. UNAIDS data demonstrate that FSW of reproductive age in low- and middle-income countries have a 24 times greater risk of HIV infection than women in the general population [2]. Therefore, FSW from sub-Saharan Africa are a key target population for ongoing efforts to reduce global HIV transmission.

The use of HIV pre-exposure prophylaxis (PrEP) improved antiretroviral access for people living with HIV, and peer-based prevention programs have all been demonstrated to decrease HIV transmission [3]. However, there are barriers to achieving these benefits in FSW populations, including low levels of engagement, health literacy, and adherence. Reduced health care utilization by FSW may be driven by legal and systemic barriers that impact ability and desire to seek care [4–9]. Thus, despite effective prevention tools, HIV rates remain high among this population and improved implementation methods are urgently needed in this population.

The criminalization of sex work in Kenya fuels a pervasive stigmatization of FSW [10]. The illegal nature of sex work creates an environment that allows for law enforcement, clients, and intimate partners to harass and assault sex workers [11], and the resultant unregulated violence towards FSW has been directly linked to increased rates of HIV acquisition [12]. Furthermore, stigmatization of FSW is also practiced by health care workers [11]. This engenders mistrust of the healthcare system in FSW, an important barrier to engagement in HIV prevention and treatment services [8].

A relatively unexplored venue for HIV prevention among FSW is the acute care setting. Emergency departments (ED) provide care to large volumes of individuals presenting for episodic care, including FSW who may rely on its services as their sole source of care. Recently, emergency medicine has been established as a rapidly expanding field in the Kenyan healthcare system [13, 14]. Common reasons for the usage of acute care medicine by FSW include physical and sexual violence, obstetrical complications, and HIV exposure [9, 15]. Acute care settings may give an opportunity to provide HIV post-exposure prevention (PEP), as well as HIV prevention education [14]. However, despite this potential, no structured guidelines exist for the management of FSW in the ED [15, 16]. The aim of this paper is to broaden our understanding of patient factors that contribute to usage of acute care services by FSW in Nairobi, Kenya, with a particular focus on the need for HIV prevention tools.

## Methods

### Participant recruitment and survey administration

An anonymous questionnaire was administered to Nairobi FSW attending Kenya AIDS Control Project (KACP) clinics during June–July 2019. All women who were currently exchanging sex for money or goods were considered FSW for the purposes of this study. The participants were eligible if they were HIV-negative and over the age of 18. The participants were recruited on a convenience basis equally across the seven clinics. The participation was voluntary, and informed consent was provided and the participants were compensated 300 KSH for their time. The study was part of a quality improvement initiative approved by the Institutional Review Boards at Kenyatta National Hospital (Kenya) and the Universities of Toronto and Manitoba (Canada). Surveys were administered in a one-on-one interview in Kiswahili or English, and responses were recorded by staff administering the survey. HIV testing was performed according to Kenyan national guidelines, with initial screening by antibody-based rapid test Determine HIV1/2 (Inverness Medical, Tokyo, Japan) and confirmation of positive tests using SD Bioline HIV1/2 (Standard Diagnostics Inc., Kyonggi Do, South Korea).

### Measures of intimate partner, client-perpetrated and other violence

An intimate partner was defined as any non-paying sexual partner, such as a husband or boyfriend. A client was defined as a partner who exchanged sex for money, rent, school fees, or other parameters. “Other” perpetrators of violence were defined as anyone other than a client or intimate partner (i.e., police, city askaris, family members). Gender-based violence was defined as any violence perpetrated against the women and manifested through acts of physical, sexual, or emotional violence. The items were structured using a modified version of the World Health Organization Violence Against Women Instrument (VAWI), which assessed experiences of 13 specific acts of physical (six items), sexual (three items), or emotional violence (four items) [17]. An extra item assessing forced sex without a condom was added to the sexual violence section, for a total of 14 items. A “yes” to at least one question in each category constituted an experience of violence, and women were dichotomized accordingly in each violence sub-group (physical, sexual, emotional). The items were asked once for the perpetration of violence by an intimate partner, once for the perpetration of violence by clients, and once for any “other” perpetrations of violence. The VAWI has demonstrated good internal validity (Cronbach’s *a* = 0.88) [18].

### Pre-exposure prophylaxis (PrEP) use

PrEP use was operationalized into four categories determined by the following question series. Participants were asked “Have you ever used PrEP?” If no, they were categorized as “never used.” If yes, the participants were asked “Are you currently taking PrEP?” If no, they were categorized as a “past user.” If yes, the participants were asked “How often do you take your PrEP pill?” If participants responded with less than six to seven times per week, they were categorized as a “current sub-optimal user.” If participants responded six to seven times per week they were categorized as a “current optimal user.”

### Depressive symptoms

Current levels of depressive symptoms were assessed using the nine-item self-reported Patient Health Questionnaire-9 (PHQ-9) to assess both diagnostic categories and severity of symptoms [19]. The participants rate the chronicity of symptoms using a four-point scale ranging from 0 (not at all) to 3 (nearly every day). The total scores of all items were summed, and the participants were categorized as meeting criteria for moderate depression (PHQ9 = 10) or not meeting criteria (PHQ-9 < 10). The PHQ-9 has been utilized widely in both research and clinical settings and possesses strong psychometric properties [20, 21]. Two large-scale validation studies in healthcare settings found excellent internal consistency for the measure (Cronbach’s *a* = 0.86 to 0.89) and support for strong test–retest reliability (*r* = 0.84) across a 48-h timeframe [22]. It has been validated among Kenyan HIV/AIDS populations (*a* = 0.78), with acceptable test–retest reliability (ICC = 0.59) [23].

### Generalized anxiety symptoms

Current levels of anxiety symptoms were similarly measured using the seven-item self-reported Generalized Anxiety Disorder7 (GAD-7) designed to assess for both presence and severity of symptoms of generalized anxiety disorder [24]. Participants rate the chronicity of symptoms using a four-point scale ranging from 0 (Not at all) to 3 (Nearly every day). Total scores of all items were summed, and the participants were categorized as meeting criteria for moderate generalized anxiety (GAD = 10) or not meeting criteria (GAD < 10). The GAD-7 has demonstrated strong psychometric properties in validation studies including excellent internal consistency (Cronbach’s *a* = 0.92) and strong test–retest reliability (*r* = 0.83). It has been validated among Kenyan HIV/AIDS populations (*a* = 0.82), with acceptable test–retest reliability (ICC = 0.70) [25].

### Emergency department health care utilization

Survey participants indicated the number of health care visits in the past year. Following this, they indicated if the visits were at an acute care or emergency department (hospital) or primary care site: pharmacy, traditional medicine, clinic, or other. If participants selected “ever” utilizing hospital services, they were classified as “acute care users.” All other participants were classified as “outpatient clinic users.” Of note, to access hospital services in Kenya, the participants must first visit the emergency department [26].

#### Statistical analysis

Categorical variables were reported as counts with percentages and analyzed with analysis of variance (ANOVA) or Fisher’s exact test whenever appropriate. Dichotomous variables were reported with percentages. Continuous variables were assessed for normal distribution using a normal probability plot and were reported as mean with standard deviation if normally distributed or median with interquartile range (IQR) if not normally distributed. Continuous variables were compared between groups using Welch’s *t* test if normally distributed or Mann–Whitney *U* test if not normally distributed. There were no multiple imputations performed.

Two negative binomial logistic regression models evaluated healthcare utilization as an outcome among the two populations. The following a priori covariates (chosen based on clinical relevance and parsimony) were included in the models: [age, years in sex work, marital status, education level, intimate partner violence (emotional, physical, and sexual), client violence (emotional, physical, and sexual), “other” violence (emotional, physical, and sexual), PrEP use, PEP use, depressive symptoms, generalized anxiety symptoms, and recent HIV-positive partner). For data acquisition and analysis, IBM SPSS version 28.0.0.0 (Armonk, New York, USA) and Stata were used.

## Results

### Participant demographics

In total, questionnaires were completed by 217 HIV-negative clinic attendees meeting the study criteria (Table 1).

**Table 1:** Population demographics

**Characteristic**	**Sub-characteristic**	**Acute Care Users**	**Outpatient Care Only Users**	***P-value***
N	-	101	116	-
# healthcare visits 1 yr	-	12.7 (2-38)	9.1 (1-53)	P<0.01
Age	-	32 (19-56)	34 (21-56)	0.15
Age Sex Work Began		23 (14-52)	25 (12-43)	0.16
# clients/week		11 (2-35)	13 (2-57)	0.13
Level of Education	Primary or less	49 (48.5%)	55 (47.4%)	0.64
	secondary	42 (41.6%)	46 (39.7%)	
	Greater than secondary	10 (9.9%)	15 (12.9%)	
Marital Status	Married	14 (13.9%)	13 (11.2%)	0.55
	single	42 (41.6%)	55 (47.4%)	
	Divorced/ widowed	45 (44.6%)	48 ( 41.45)	
IPV	No IP	24	29	-
	emotional	52 (67.5%)	56 (48.3%)	0.64
	physical	44 (57.1%)	50 (43.1%)	0.59
	sexual	48 (62.3%)	45 (38.8%)	0.93
Client Perpetrated Violence	emotional	76 (75.2%)	83 (71.6%)	0.56
	physical	50 (49.5 %)	65 (56.0%)	0.37
	sexual	74 (73.3%)	75 (64.7%)	0.16
Other** Violence	emotional	48 (47.5%)	58 (50.0%)	0.57
	physical	39 (38.6%)	47 (40.5%)	0.61
	sexual	26 (25.7%)	23 (19.8%)	0.37
PHQ9score	<9 (mild)	41 (40.6%)	53 (45.7%)	0.37
	10-14 (moderate)	36 (35.6%)	40 (34.5%)	
	15+ (severe)	24 (23.8%)	23 (19.85)	
GAD7	mild	51 (50.5%)	61 (52.6%)	0.94
	mod	35 (34.7 %)	37 (31.9%)	
	severe	15 (14.9%)	18 (15.5%)	
PreP use	Current optimal	49 (48.5%)	57 (49.1%)	0.03
	Poor use, non use	52 (51.5%)	59 (50.9%)	
Birth control	Condoms only	55 (54.5%)	49 (42.2%)	0.18
	OCP	11 (10.9%)	12 (10.3%)	
	depo/IUD	35 (34.7%)	55 (47.4%)	
Physician trust	low	16 (15.8%)	12 (10.3%)	0.12
	ambiguous	21 (20.8%)	19 (16.4%)	
	high	64 (63.4%)	85 (73.3%)	
Self-rated health status	good	39 (38.6%)	61 (52.6%)	0.01
	ambiguous	36 (35.6%)	37 (31.9%)	
	poor	26 (25.7%)	18 (15.5%)	
Substance Use	alcohol	69 (68.3%)	80 (69%)	0.54
	other	45 (44.6%)	44 (37.9%)	0.31
Pep Use	-	52 (51.5%)	57 (49.1%)	0.77
Lube Use	-	66 (65.3%)	81 (69.8%)	0.43
HIV test within 3 months	-	89 (88.1%)	112 (96.6%)	0.05
Condom use with 100% of clients	-	76 (75.2%)	88 (75.9%)	0.78
Previously treated STI	-	67 (66.3%)	73 (62.9%)	0.54
HIV+ Client in prior 6 Months	-	66 (65.3%)	51 (44%)	0.03

Continuous variables are reported as median (interquartile range) unless otherwise specified

*PHQ9* Patient Health Qusetionnaire-9, *GAD7* Generalized Anxiety Disorder-7, *PrEP* pre-exposure prophylaxis, **Other is defined as any other perpetrator of abuse other than an IP or client, most frequently documented as Law-Enforcement

One hundred one participants were defined as “acute care users,” and 116 were defined as the control population. The mean number of health care visits by acute care users was 12.7 ± 8.5, which was higher than the 9.1 ± 7.0 visits reported by controls (*p* < 0.05). Among acute care users, 86 (85%) reported any form of intimate partner violence, and 93 (92%) reported any form of client perpetrated violence (Table 1), not statistically different from the control population. Only 64 (64%) of acute care users reported high physician trust. The average PHQ9 and GAD 7 scores for acute care users were 11.5 ± 5.2 and 9.2 ± 4.5, respectively. Finally, 49 (48.5%) and 57 (49.1%) of acute care users and control users, respectively, were currently taking PreP as prescribed.

### Violence

Violence was commonly reported, although the prevalence of all forms of violence did not differ between acute care users and the control population. Multivariate analysis (Table 2) indicated that client perpetrated physical violence was associated with a decrease in health care visits among acute care users (OR 0.65 [0.51–0.82], *p* < 0.01). Increases in health care visits corresponded with having experienced client perpetrated sexual violence among acute care users (OR 2.2 [1.64–2.94], *p* < 0.01). IPV was found to have no association with the number of health care visits (Table 2). Neither of these variables was associated with the number of health care visits in the control population (Table 3).

**Table 2:** Factors associated with increase healthcare use among FSW who utilized emergency departments

Parameter	Sub-group	OR	CI	*P* value
Marital status	Divorced/widowed	1	-	-
	Single	0.80	0.64–0.98	0.05
	Married	0.56	0.41–0.77	< 0.01
Education	Post secondary (ref)	1	-	-
	Secondary	0.83	0.57–1.20	0.33
	Primary or less	0.66	0.45–0.94	0.03
Prep risk	High (ref)	1	-	-
	Low	1.54	1.25–1.91	< 0.01
Client physical violence	No (ref)	1	-	-
	Yes	0.65	0.51–0.82	< 0.01
Client sexual violence	No (ref)	1	-	-
	Yes	2.2	1.64–2.94	< 0.01
Other physical violence	No (ref)	1	-	-
	Yes	1.39	1.10–1.75	< 0.01
Physician trust	High (ref)	1	-	-
	Ambiguous	0.96	0.73–1.25	0.75
	Low	1.33	1.06–1.76	0.05
GAD7	High (ref)	1	-	-
	Mod	1.36	1.00–1.85	0.05
	Low	1.61	1.18–2.20	< 0.01
HIV pos partner	No (ref)	1	-	-
	Yes	1.35	1.02–1.69	< 0.01
PEP use	No (ref)	1	-	-
	Yes	0.77	0.62–0.95	0.014
Age		1.13	0.99–1.028	0.06
IP sexual violence		No impact		

Negative log binominal analysis assessing how FSW-specific characteristics influence health care utilization among those who endorsed seeking healthcare at both ED’s and outpatient clinics

**Table 3:** Factors associated with increase healthcare use among FSW who utilized outpatient clinics

Parameter	Sub-group	OR	CI	*P* value
Marital status	Divorced/widowed	1	-	-
	Single	1.24	0.95–1.62	0.12
	Married	0.91	0.61–1.62	0.64
Education	Post-secondary (ref)	1	-	-
	Secondary	0.69	0.47–1.00	0.05
	Primary or less	0.75	0.51–1.11	0.15
Prep risk	High (ref)	1	-	-
	Low	1.76	1.40–2.22	< 0.01
Client physical violence	No (ref)	1	-	-
	Yes	0.93	0.69–1.24	0.41
Client sexual violence	No (ref)	1	-	-
	Yes	1.15	0.82–1.62	0.41
Other physical violence	No (ref)			
	Yes	1.01	0.70–1.45	0.95
Physician trust	High (ref)	1	-	-
	Ambig	0.92	0.65–1.32	0.65
	Low	0.72	0.49–1.07	0.10
GAD7	High (ref)	1	-	-
	Mod	1.24	0.87–1.76	0.24
	Low	0.92	0.66–1.28	0.91
HIV pos partner	No (ref)			
	Yes	1.01	0.79–1.29	0.92
PEP use	No (ref)	0.85	0.66–1.10	0.21
	Yes			
Age		1.02	1.00–1.03	0.06
IP sexual violence	No violence	1	-	-
	Violence	0.67	0.48–0.94	0.019
	No partner	0.79	0.56–1.12	0.18

Negative log binominal analysis assessing how FSW-specific characteristics influence health care utilization among those who endorsed seeking healthcare only at outpatient clinics

### Anxiety and depression scores

There was no difference in GAD7 and PHQ9 scores between acute care users and control participants, and depression did not correlate with health care utilization in either cohort. High levels of anxiety were associated with decreased health care visits among acute care users (low anxiety OR 1.61 (1.18–2.20), *p* < 0.01). The relationship was not replicated in the control population (0.92 (0.66–1.28), *p* = 0.91).

### Sex practices

Sexual and health care practices (e.g., lubricant use, HIV testing, PEP use, contraception) did not differ significantly between the control group and group of interest. High PrEP risk (defined as lack of or poor PrEP adherence) was found to be correlated with decreased health care utilization in both acute and control populations. Individuals with lower PrEP risk (proper PrEP use and adherence) were more likely to seek out care (acute use OR = 1.54 (1.25–1.91), *p* < 0.01, control cohort OR = 1.76 (1.40–2.22), *p* < 0.01). Having an HIV-positive partner in the past 6 months was correlated with increased acute care utilization (OR = 1.35 (1.02–1.69), *p* < 0.01), but not clinic use by the control population.

## Discussion

**Fig. 1:**
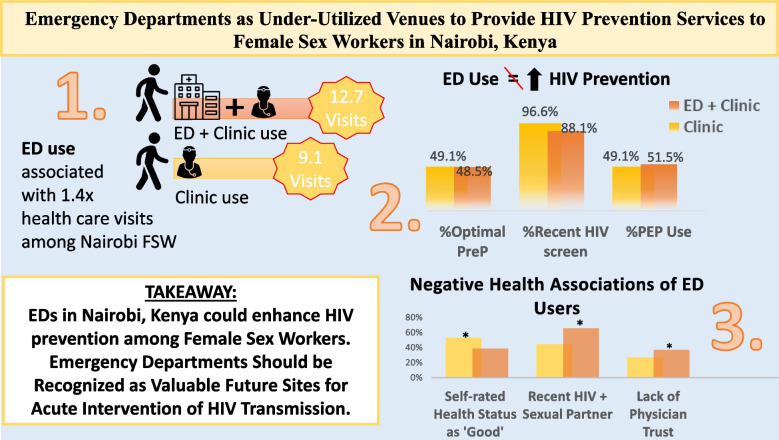
ED use facilitates HCP interactions with FSW, no improvement in HIV prevention. Legend: *HCP* healthcare provider, *FSW* female sex worker, *ED* emergency department. “ED + Clinic” defined as FSW whom accessed health care through emergency departments and outpatient sex worker-specific clinics. “Clinic” represented FSW who utilized only clinics when accessing care

There are currently disproportionately high rates of HIV incidence and prevalence among FSW in sub-Saharan Africa. Interestingly, little research has been done to characterize how ED in these countries may be utilized to support HIV prevention in FSW populations. Our goal was to investigate the relationship between emergency department utilization and FSW characteristics. We found that despite the use of greater services over a 1-year period, FSW’s who utilized both emergency departments and clinics as opposed to outpatient clinics alone reported no improvements in HIV prevention tools usage, such as PEP and PrEP uptake (Fig. 1). Moreover, the FSW population who utilize emergency departments reported lower HIV testing rates, lower-self reported health status, and greater exposure to HIV-positive partners than clinic users. These findings suggest the need for optimizing emergency departments as HIV intervention sites for FSW, as can be used to inform policy aiming to utilize these settings for HIV prevention strategies.

Despite ongoing education and improved access, the proportion of our study participants who engaged in safe sex practices was lower than anticipated. Roughly half of participants reported no or inappropriate PrEP usage. Poor PrEP use was associated with decreased healthcare utilization in both the acute care population and outpatient clinic population, suggesting FSW are unmotivated to seek care when exposures occur [27]. Additionally, the results of our study demonstrate that acute care users engaged in more health care visits over the period of study, but did not report improved access to contraception or HIV prevention tools. We know the opposite to be true in locations such as North America and Europe, where ED used by marginalized populations increases uptake of HIV prevention tools [28, 29]. This data suggests emergency departments in Kenya are not effectively addressing FSW-specific health care needs when this population uses these services. Emergency departments should be aware that a low percentage of sex workers is taking PrEP effectively or accessing other HIV prevention services, as to utilize emergency department encounters as educational opportunities. While our study was limited to HIV seronegative individuals, the results are supportive of the ED as a unique venue for increased HIV testing, PrEP access, and education.

The associations between sex work and gender-based violence have been well documented [30, 31]. Our study found that client-perpetrated sexual violence was associated with increased healthcare utilization; however, client perpetrated physical violence was associated with decreased health care usage among acute care users. Given that sexual assault is not a sex worker-specific occurrence among Kenyan women, it is reasonable to infer that FSW use these services without needing to disclose the nature of their work. However, the criminalization of sex work in Kenya creates an environment in which violence is tolerated, making physical violence by a client much more complicated to report [32]. Reasons for lack of reporting include fear of punishment and lack of guaranteed safety after reporting, shame, financial barriers, lack of access to services, and distrust of healthcare workers [30, 33, 34]. Following, lack of reporting directly impacts HIV transmission, with FSW being over seven times more likely to contract HIV in a country where sex work is illegal [32]. There are excellent examples of how emergency departments have begun to integrate violence care guidelines into their practice which have led to improved provider comfort in delivery of care and patient satisfaction [35–38]. However, so far, these guidelines only address gender-based violence among the general population and thus fail to address sex worker-specific needs. Specific interventions taking into consideration the needs of local populations must be implemented among sub-Saharan FSW populations to directly combat HIV acquisition amongst this cohort.

Further, our study found that high levels of anxiety were associated with decreased acute healthcare utilization but not decreased clinic service utilization. A likely contributor to this decrease is due to the experienced or anticipated sigma from health care workers, as opposed to the dedicated FSW clinics designed for use by these populations. Discrimination and perceived stigma from health care workers catalyze avoidance of medical services among FSW, subsequently depriving them of access to health services [8, 32, 34]. This is counterproductive towards government efforts to reduce HIV rates, as the emergency department could serve as an excellent touch point in providing HIV preventative care in these patients. As emergency medicine care in Kenya expands, efforts can be made to shift Kenyans’ negative attitudes towards sex workers through anti-discrimination policies and sensitivity-based training [11, 39–41].

Although our study contributes important findings to the limited existing literature on emergency department utilization among FSW, several limitations exist. A major limitation to our study is that we do not have access to information about individual health care visits and are operating under assumptions that many of the visits are due to FSW specific needs. Further, questionnaires were only administered to FSW attending KACP clinics, and data could only be assessed from those attendees who agreed to participate. Therefore, based on the limits of convenience sampling, it remains unknown to what extent the results apply to FSW not in care, who accessed other services, or who declined the questionnaire. In addition, the use of a facility-based convenience sample may have led to our study having an enriched enrolment of FSW taking PrEP or FSW more engaged in health care. Further, our study is cross-sectional in design, and so, the direction of causation cannot be defined for the associations that we describe. Finally, reporting bias (overreporting of PrEP and/or underreporting of violence) may have skewed associations. Our results may have been confounded by other unmeasured factors, such as adverse childhood experiences, violence predating the 12-month time frame, indirect cost of accessing services, or other non-measured reasons for non-adherence to PrEP such as fear of side effects. Nonetheless, our results have clear implications for quality improvement within the ED and merit broader consideration within FSW clinics elsewhere.

## Conclusions

Across North America and Europe, the role of emergency medicine in the prevention of HIV among vulnerable populations has been long established [14]. In contrast, there are only limited reports of programs focusing on HIV prevention among vulnerable FSW populations in the ED in low-resource countries such as Kenya [14, 15]. In an effort to better understand this population and their use of emergency departments, our study demonstrates that ED utilization among FSW is not successfully increasing access to HIV prevention with the same success as North American and European EDs. The results of this study should be used to guide policy design, health care provider training, and facility preparedness to support improved relationships between FSW and ED visits in attempts to curb HIV transmission.

### Outcomes and impact

- (1) Educate clinicians on sex worker characteristics and the vulnerability factors clinicians must consider.
- (2) Advance a research and culturally competent clinical training agenda that can optimize health care engagement and utilization within the sex work community.

## Data Availability

The datasets generated and/or analyzed during the current study are not publicly available due patient privacy reasons but are available from the corresponding author on reasonable request.

## Abbreviations

FSW: Female sex workers
GAD7: Generalized Anxiety Disorder-7
IPV: Intimate partner violence
KACP: Kenya Aids Control Project
PHQ9: Patient Health Questionnaire-9
PrEP: Pre-exposure prophylaxis
VAWI: Violence Against Women Instrument

## References

[1] Wanyenze RK, Musinguzi G, Kiguli J (2017). When they know that you are a sex worker, you will be the last person to be treated: perceptions and experiences of female sex workers in accessing HIV services in Uganda. BMC Int Health Hum Rights.

[2] UNAIDS global AIDS update 2021. Confronting inequalities: lessons for pandemic responses from 40 years of AIDS. Available at: https://www.unaids.org/sites/default/files/media_asset/2021-global-aids-update_en.pdf. 2021. Accessed 27 July 2023.

[3] Green SN (2017). Seizing the moment. Sur.

[4] Napierala S, Chabata ST, Davey C, et al. Engagement in HIV services over time among young women who sell sex in Zimbabwe. PLoS One. 2022;17(6):e0270298. 10.1371/journal.pone.0270298 PMC9239457 35763532

[5] Witte SS, Filippone P, Ssewamala FM, et al. PrEP acceptability and initiation among women engaged in sex work in Uganda: implications for HIV prevention. eClinicalMedicine. 2022;44:101278. 10.1016/j.eclinm.2022.101278. 10.1016/j.eclinm.2022.101278 PMC8808048 35128367

[6] Eshikumo P, Awuor P, Blanco N, et al. Factors associated with retention in HIV prevention and treatment clinical services among female sex workers enrolled in a Sex Workers’ Outreach Program (SWOP) in Nairobi. Kenya AIDS Behav. Published online 2022. 10.1007/s10461-022-03654-0. 10.1007/s10461-022-03654-0 PMC10256564 35299260

[7] Ghimire L, Smith WCS, Van Teijlingen ER. Utilisation of sexual health services by female sex workers in Nepal. BMC Health Serv Res. 2011;11. 10.1186/1472-6963-11-79. 10.1186/1472-6963-11-79 PMC3107775 21501473

[8] Hall BJ, Sou KL, Beanland R, et al. Barriers and facilitators to interventions improving retention in HIV care: a qualitative evidence meta-synthesis. AIDS Behav. Published online 2017. 10.1007/s10461-016-1537-0. 10.1007/s10461-016-1537-0 PMC5332336 27582088

[9] Lafort Y, Greener R, Roy A, et al. Sexual and reproductive health services utilization by female sex workers is context-specific: results from a cross-sectional survey in India, Kenya, Mozambique and South Africa. Reprod Health. Published online 2017. 10.1186/s12978-017-0277-6. 10.1186/s12978-017-0277-6 PMC5247811 28103896

[10] Scorgie F, Chersich MF, Ntaganira I, Gerbase A, Lule F, Lo YR (2012). Socio-demographic characteristics and behavioral risk factors of female sex workers in sub-Saharan Africa: a systematic review. AIDS Behav.

[11] Mbote DK, Nyblade L, Kemunto C, et al. Police discrimination, misconduct, and stigmatization hhr_final_logo_alone.Indd 1 of female sex workers in kenya: associations with delayed and avoided health care utilization and lower consistent condom use. Health Hum Rights. 2020;22(2):199–212. PMC7762893 33390707

[12] Shannon K, Strathdee SA, Goldenberg SM (2015). Global epidemiology of HIV among female sex workers: influence of structural determinants. Lancet.

[13] Lee JA, Wanjiku G, Nduku N (2022). The status and future of emergency care in the Republic of Kenya. Afr J Emerg Med.

[14] Waxman MJ, Muganda P, Carter EJ, Ongaro N. The role of emergency department HIV care in resource-poor settings: lessons learned in western Kenya. Int J Emerg Med. Published online 2008. 10.1007/s12245-008-0065-8. 10.1007/s12245-008-0065-8 PMC2657254 19384648

[15] Waxman MJ, Kimaiyo S, Ongaro N, Wools-Kaloustian KK, Flanigan TP, Carter EJ. Initial outcomes of an emergency department rapid HIV testing program in western Kenya. AIDS Patient Care STDS. Published online 2007. 10.1089/apc.2007.0075. 10.1089/apc.2007.0075 18154494

[16] Emergency Medicine Kenya Foundation. Emergency care algorithms 2023. 2023. Available at: https://www.emergencymedicinekenya.org/algorithms/. Accessed 27 July 2023.

[17] Garcia-Moreno C, Jansen HA, Ellsberg M, Heise L, Watts CH. Prevalence of intimate partner violence: findings from the WHO multi-country study on women’s health and domestic violence. Lancet. Published online 2006. 10.1016/S0140-6736(06)69523-8. 10.1016/S0140-6736(06)69523-8 17027732

[18] Nybergh L, Taft C, Krantz G. Psychometric properties of the WHO violence against women instrument in a female population-based sample in sweden: a cross-sectional survey. BMJ Open. Published online 2013. 10.1136/bmjopen-2012-002053. 10.1136/bmjopen-2012-002053 PMC3664346 23793692

[19] Willie TC, Overstreet NM, Sullivan TP, Sikkema KJ, Hansen NB. Barriers to HIV medication adherence: examining distinct anxiety and depression symptoms among women living with HIV who experienced childhood sexual abuse. Behav Med. Published online 2016. 10.1080/08964289.2015.1045823. 10.1080/08964289.2015.1045823 PMC4710561 26010763

[20] Löwe B, Kroenke K, Herzog W, Gräfe K. Measuring depression outcome with a brief self-report instrument: sensitivity to change of the Patient Health Questionnaire (PHQ-9). J Affect Disord. Published online 2004. 10.1016/S0165-0327(03)00198-8. 10.1016/S0165-0327(03)00198-8 15183601

[21] Kroenke K, Spitzer RL. The PHQ-9: a new depression diagnostic and severity measure. Psychiatr Ann. Published online 2002. 10.3928/0048-5713-20020901-06.

[22] Kroenke K, Spitzer RL, Williams JB (2001). The PHQ-9: validity of a brief depression severity measure. J Gen Intern Med..

[23] Monahan PO, Shacham E, Reece M, et al. Validity/reliability of PHQ-9 and PHQ-2 depression scales among adults living with HIV/AIDS in Western Kenya. J Gen Intern Med. Published online 2009. 10.1007/s11606-008-0846-z. 10.1007/s11606-008-0846-z PMC2629000 19031037

[24] Spitzer RL, Kroenke K, Williams JBW, Löwe B. A brief measure for assessing generalized anxiety disorder: the GAD-7. Arch Intern Med. Published online 2006. 10.1001/archinte.166.10.1092. 10.1001/archinte.166.10.1092 16717171

[25] Nyongesa MK, Mwangi P, Koot HM, Cuijpers P, Newton CRJC, Abubakar A. The reliability, validity and factorial structure of the Swahili version of the 7-item generalized anxiety disorder scale (GAD-7) among adults living with HIV from Kilifi. Kenya Ann Gen Psychiatry. Published online 2020. 10.1186/s12991-020-00312-4. 10.1186/s12991-020-00312-4 PMC7594456 33133222

[26] Matifary CR, Wachira B, Nyanja N, Kathomi C. Reasons for patients with non-urgent conditions attending the emergency department in Kenya: a qualitative study. African J Emerg Med. Published online 2021. 10.1016/j.afjem.2020.09.004. 10.1016/j.afjem.2020.09.004 PMC7910189 33680731

[27] Restar AJ, Tocco JU, Mantell JE, et al. Perspectives on HIV pre-and postexposure prophylaxes (prep and pep) among female and male sex workers in mombasa, Kenya: implications for integrating biomedical prevention into sexual health services. AIDS Educ Prev. Published online 2017. 10.1521/aeap.2017.29.2.141. 10.1521/aeap.2017.29.2.141 PMC5706461 28467163

[28] Branson BM, Handsfield HH, Lampe MA, et al. Revised recommendations for HIV testing of adults, adolescents, and pregnant women in health-care settings. MMWR Recomm Rep. Published online 2006. 10.1016/j.annemergmed.2007.03.001. 16988643

[29] Menchine M, Zhou M, Lotfipour S, Chakravarthy B. Moving beyond screening: How emergency departments can help extinguish the HIV/AIDS epidemic. West J Emerg Med. Published online 2016. 10.5811/westjem.2016.1.29100. 10.5811/westjem.2016.1.29100 PMC4786231 26973737

[30] Micheni M, Rogers S, Wahome E, et al. Risk of sexual, physical and verbal assaults on men who have sex with men and female sex workers in coastal Kenya. Aids. 2015;29(0 3):S231-S236. 10.1097/QAD.0000000000000912. 10.1097/QAD.0000000000000912 PMC4706373 26562812

[31] Leis M, McDermott M, Koziarz A, et al. Intimate partner and client-perpetrated violence are associated with reduced HIV pre-exposure prophylaxis (PrEP) uptake, depression and generalized anxiety in a cross-sectional study of female sex workers from Nairobi, Kenya. J Int AIDS Soc. 2021;24(S2). 10.1002/jia2.25711. 10.1002/jia2.25711 PMC8222843 34164924

[32] Lyons CE, Schwartz SR, Murray SM, et al. The role of sex work laws and stigmas in increasing HIV risks among sex workers. Nat Commun. 2020;11(1). 10.1038/s41467-020-14593-6. 10.1038/s41467-020-14593-6 PMC7028952 32071298

[33] Illangasekare S, Burke J, Chander G, Gielen A (2013). The syndemic effects of intimate partner violence, HIV/AIDS, and substance abuse on depression among low-income Urban women. J Urban Heal.

[34] Hatcher AM, Smout EM, Turan JM, Christofides N, Stöckl H. Intimate partner violence and engagement in HIV care and treatment among women: a systematic review and meta-analysis. AIDS. Published online 2015. 10.1097/QAD.0000000000000842. 10.1097/QAD.0000000000000842 26353027

[35] Ranney ML, Rennert-May E, Spitzer R, Chitai MA, Mamlin SE, Mabeya H. A novel ED-based sexual assault centre in western Kenya: description of patients and analysis of treatment patterns. Emerg Med J. Published online 2011. 10.1136/emj.2010.096412. 10.1136/emj.2010.096412 20947922

[36] Wangamati CK, Gele AA, Sundby J. Post rape care provision to minors in Kenya: an assessment of health providers’ knowledge, attitudes, and practices. J Interpers Violence. Published online 2020. 10.1177/0886260517696863. 10.1177/0886260517696863 29294671

[37] Krolikowski AM, Koyfman A. Emergency centre care for sexual assault victims. African J Emerg Med. Published online 2012. 10.1016/j.afjem.2011.12.005.

[38] Silva-Nash JR, Bordelon S, Dare RK, Searcy S. 986. Improvement in administration of HIV post-exposure prophylaxis in the emergency department following sexual assault. Open Forum Infect Dis. Published online 2020. 10.1093/ofid/ofaa439.1172.

[39] Wanjiru R, Nyariki E, Babu H (2022). Beaten but not down! Exploring resilience among female sex workers (FSWs) in Nairobi. Kenya BMC Public Health.

[40] van der Elst EM, Gichuru E, Omar A, et al. Experiences of Kenyan healthcare workers providing services to men who have sex with men: qualitative findings from a sensitivity training programme. J Int AIDS Soc. Published online 2013. 10.7448/IAS.16.4.18741. 10.7448/IAS.16.4.18741 PMC3852126 24321109

[41] Evens E, Lanham M, Santi K, et al. Experiences of gender-based violence among female sex workers, men who have sex with men, and transgender women in Latin America and the Caribbean: a qualitative study to inform HIV programming. BMC Int Health Hum Rights. Published online 2019. 10.1186/s12914-019-0187-5. 10.1186/s12914-019-0187-5 PMC6399914 30832664

